# Essential oil variation in wild populations of *Artemisia saharae* (Asteraceae) from Tunisia: chemical composition, antibacterial and antioxidant properties

**DOI:** 10.1186/s40529-014-0076-0

**Published:** 2014-12-10

**Authors:** Sami Zouari, Imen Ayadi, Nahed Fakhfakh, Hamida Jdir, Latifa Aloui, Mohamed Kossentini, Ahmed Rebai, Nacim Zouari

**Affiliations:** 1 Laboratoire de Chimie Appliqué: Hétérocycles, Corps gras et Polymères, Faculté des Sciences de Sfax, Université de Sfax, Sfax, 3000 Tunisia; 2 Laboratoire de Microorganismes et de Biomolécules, Equipe de criblage moléculaire et cellulaire, Centre de Biotechnologie de Sfax, B.P. 1177, Sfax, 3018 Tunisia; 3 Institut Supérieur de Biologie Appliquée de Médenine, Université de Gabès, Médenine, 4119 Tunisia; 4 Laboratoire de Biochime et de Génie Enzymatique des Lipases, Université de Sfax, Ecole Nationale d’Ingénieurs de Sfax, Sfax, 3038 Tunisia

**Keywords:** Artemisia saharae, White wormwood, Essential oil, Discriminant analysis, Antimicrobial activity, Antioxidant activity

## Abstract

**Background:**

*Artemisia saharae* Pomel is a new taxon of *Artemisia herba-alba* Asso (Asteraceae) which is endemic to Tunisia and Algeria. This shrub, commonly known as white wormwood or desert wormwood, is largely used in folk medicine and as a culinary herb. The bulks aromatic plants come from wild populations whose essential oils compositions as well as their biological properties are severely affected by several factors such as geographic conditions. Therefore, the aim of the present work is to provide more information about the influence of altitude variation on the essential oil composition, antimicrobial and antioxidant properties of *Artemisia saharae* growing wild in the same geographical area.

**Results:**

Essential oils were extracted by hydrodistillation of leaves and flowers of the plant collected from seven different altitudes of the Baten Zamour region (southwest of Tunisia). The highest essential oil yields (2.70-2.80%) were obtained for populations of high altitudes.

Seventy-five compounds, representing 92.78 to 96.95% of the total essential oils, were separated and identified. Essential oils were characterized by very high percentage of oxygenated monoterpenes (52.1–72.6%) which constituted the predominant class. From the analyzed populations, the major compounds (>7%) were α-thujone, β-thujone, chrysanthenone, camphor, chrysanthenyl acetate, and sabinyl acetate. Sabinyl acetate which was detected in some populations at relatively high percentages (7.7–10.8%) seems to be characteristic to Southern Tunisian *A. saharae*. The studied essential oil showed a chemical diversity depending on the population altitude as revealed by linear discriminant and cluster analyses.

**Conclusions:**

Three population groups associated with altitudinal levels were distinguished. It is worthy to note that the most discriminating compounds of chemical groups were the minor ones. Despite the high variation of essential oil compositions, the high altitude population did not affect severely the antibacterial activity against the most tested strains. Altitude seems to be an important factor influencing the yield and the chemical profile of *Artemisia saharae* essential oils. Knowledge of the chemical composition of essential oils in relation to environmental factors is a very important quality criterion for their marketing and contributes to their valorization as functional ingredient in food technology.

**Electronic supplementary material:**

The online version of this article (doi:10.1186/s40529-014-0076-0) contains supplementary material, which is available to authorized users.

## Background

In recent years, there has been a growing concern about the potentially adverse effects of chemical additives used in food industry and medicine. Therefore, there was a marked interest for natural products, considered to have very few side effects, as compared to synthetic chemicals. Consequently, the commercial development of medicinal plants and their derivatives, such as essential oils, as new sources of bioactive products to enhance human health and food preservation is of prime importance. Essential oils, formed by medicinal and aromatic plants as secondary metabolites, were very heterogeneous mixtures that may contain many compounds at different concentrations. However, several factors, namely climatic, geographic conditions, and ontogeny of collected plants may severely affect essential oil yield, their composition, and their biological properties. Thus, studies of chemical variability of essential oil in relation to environmental factors might provide information on what determines its chemical polymorphism. In addition, knowledge of the essential oils chemotypes is a very important quality criterion for their marketing and contributes to their commercialization as functional ingredients in food technology or in phytopharmacy (Zouari, [2013]).

Recently, Le Floc’h et al. ([2010]) reported that *Artemisia saharae* Pomel is a new taxon of *Artemisia herba-alba* Asso (Asteraceae) which is endemic to Tunisia and Algeria. This shrub, commonly known as white wormwood or desert wormwood (Arabic name chih), was widely used in traditional medicine against diabetes, bronchitis, diarrhea, neuralgias, and hypertension (Tahraoui et al*.*[2007]). The antioxidant, antihypertensive, antibacterial, antileshmanial, anthelmintic and antispasmodic activities of *A. herba-alba* essential oils have been reported (Yashphie et al*.*[1987]; Hatimi et al*.*[2001]; Zouari et al*.*[2010]). Previous studies assessed on the composition variability of *A. herba-alba* essential oils have essentially considered the effect of the geographical locations and consequently different climatic conditions (Mighri et al. [2010]; Belhattab et al*.*[2014]). However, there is little information about the influence of altitude variation on the essential oil composition of *A. herba-alba* growing in the same geographical area. In fact, altitude is one of the abiotic stresses which is associated with alterations in a number of environmental factors (Kofidis et al. [2003]).

In Baten Zamour region (Gafsa, southwest of Tunisia), *A. saharae* populations were found in different altitudes. Therefore, the aim of the present work is to provide more information on the chemical diversity of *A. saharae* volatiles collected from seven different altitudinal wild populations. The chemical variability of the essential oils among samples was assessed by linear discriminant analysis and UPGMA (unweighted pair-group method with averaging) cluster analysis. Besides, influence of altitude on yield, and antibacterial and antioxidant properties of essential oils was also assessed.

## Methods

### Populations analyzed and sampling

The seven populations of *A. saharae* Pomel (= *A. herba-alba* Asso) collected from the same locality and from different altitudes were reported in Table 1. Five individuals (*n* = 5) from each population were sampled over the entire population area at the flowering stage (November 2012). The distance between individuals exceeded 20 m, to avoid collection from close parents. After that, the fresh vegetable matter was dried in the shade, until constancy of the mass (20 days). Separated from stems, aerials parts were subjected for essential oil extraction.

**Table 1:** **Location of the analyzed populations of** ***A. herba-alba***

Population^a^	Latitude	Longitude	Altitude (m)
1	34° 21’ 03” N	009° 23’ 31” E	194
2	34° 20’ 58” N	009° 23’ 30” E	206
3	34° 20’ 55” N	009° 23’ 30” E	219
4	34° 20’ 53” N	009° 23’ 31” E	230
5	34° 20’ 51” N	009° 23’ 30” E	240
6	34° 21’ 59” N	009° 12’ 55” E	361
7	34° 24’ 41” N	009° 13’ 31” E	841

^a^The numbering refers to the *A. herba-alba* populations.

### Essential oil extraction

The dry matter was submitted to hydrodistillation for 4 h, using a Clevenger-type apparatus. Each essential oil was dried over anhydrous sodium sulphate and stored in sealed vials protected from light at −20°C until analysis.

### Essential oil analyses

#### Gas chromatography (GC)

A Hewlett-Packard 5890 series II gas chromatograph equipped with HP-5MS capillary column 30 m × 0.25 mm i.d., film thickness 0.25 μm; Hewlett-Packard) and connected to a flame ionization detector (FID) was used. The column temperature was programmed at 50°C for 1 min, then 7°C/min to 250°C, and then left at 250°C for 5 min. The injection port temperature was 240°C and that of the detector 250°C (split ratio: 1/60). The carrier gas was helium (99.995% purity) with a flow rate of 1.2 ml/min and the analysed sample volume was 2 μl. Percentages of the constituents were calculated by electronic integration of FID peak areas, without the use of response factor correction. Mean percentage of compounds in *A. herba-alba* essential oils represented the average calculated on five individuals (*n* = 5). Retention indices (RI) were calculated for separate compounds relative to (C_7_ – C_25_) n-alkanes mixture (Aldrich Library of Chemicals Standards) (Kovàts [1958]).

#### Gas chromatography/Mass spectrometry (GC/MS)

The isolated volatile compounds were analysed by GC/MS, using an Agilent Technologies 6890 N gas chromatograph. The fused HP-5MS capillary column (the same as that used in the GC/FID analysis) was coupled to an Agilent Technologies 5973B mass-spectrometer (Hewlett-Packard, Palo Alto, CA, USA). The oven temperature was programmed at 50°C for 1 min, then 7°C/min to 250°C, and then left at 250°C for 5 min. The injection port temperature was 250°C and that of the detector was 280°C (split ratio: 1/100). The carrier gas was helium (99.995% purity) with a flow rate of 1.2 ml/min. The mass spectrometer conditions were as follow: ionization voltage, 70 eV; ion source temperature, 150°C; electron ionization mass spectra were acquired over the mass range 50–550 m/z.

### Volatile compounds identification

The essential oil compounds of *A. herba-alba* were identified by comparing the mass spectra data with spectra available from the Wiley 275 mass spectra libraries (software, D.03.00). Further identification confirmations were made referring to retention indices (RI) data generated from a series of known standards of n-alkanes mixture (C_7_ – C_25_) (Kovàts [1958]) and to those previously reported in the literature (Adams [2001]; Zouari et al. [2010]).

### Antioxidant activities

The metal (Fe^2+^) chelating and DPPH radical-scavenging activities of essential oils were measured as previously described (Dinis et al. [1994]; Kirby and Schmidt [1997]). Metal (Fe^2+^) chelating activity was presented by IC_50_ values, defined as the concentration of the extract needed to chelate 50% of Fe^2+^ present in the test solution. Therefore, IC_50_ values were calculated from the graph of chelating percentages against extract concentration. Lower IC_50_ values reflected better chelating activity. Ethylenediaminetetraacetic acid (EDTA) and butylhydroxyanisole (BHA) were used as positive controls for metal (Fe^2+^) chelating and DPPH radical-scavenging activities, respectively.

### Antibacterial activity

#### Bacterial strains

Antibacterial activities of *A. herba-alba* essential oils were tested against 6 strains of bacteria: three Gram-negative (*Escherichia coli*, *Klebsiella pneumoniae*, and *Salmonella typhimurium*) and three Gram-positive (*Bacillus cereus*, *Enterococcus faecalis*, and *Micrococcus luteus*). Microorganisms were obtained from the culture collection of the Laboratory of Enzyme Engineering and Microbiology (“Ecole Nationale d’Ingénieurs de Sfax”, Tunisia).

### Determination of the minimum inhibitory concentration (MIC)

MICs values, which represented the lowest essential oil concentration that preventing visible growth of microorganisms, were determined as previously described (Ben Bnina et al. [2009]). All tests were performed in Mueller–Hinton broth (MHB) supplemented with 5% dimethylsulfoxide (DMSO). Bacterial strains were cultured overnight in MHB medium at 37°C. Tubes of MHB containing various concentrations of essential oils were inoculated with 10 μl bacterial inoculums adjusted to 10^6^ cfu/ml of bacteria cells. Then, they were incubated under shaking conditions (200 rpm) for 24 h at 37°C. Control tubes without tested samples were essayed simultaneously. All tests were carried out for five sample replications and the results were averaged.

### Statistical analyses

The distribution of the 75 compounds identified from the essential oil was checked by a descriptive statistical analysis using the SPSS software for Windows™ (version 17, SPSS Inc., Chicago, IL, USA). The percentages of compounds were transformed using the arcsine transformation in order to improve the distribution property. However, this transformation did not yield satisfactory results for 18 variables. Therefore, for compounds having skewed distributions, a non parametric one-way analysis of variance Kruskal-Wallis test (SPSS software, 17.0) was performed. The chemical population structure assessed by Linear Discriminant Analysis (LDA) and dendrogram analysis were performed as previously described (Zouari et al. [2012]). Duncan’s multiple range test (*p* < 0.05) was used to compare the averages of essential oil yields and the metal (Fe^2+^) chelating and the DPPH radical-scavenging activities among populations. Correlation and the covariance analysis between the MICs values of strains and the populations’ altitudes were performed using the SPSS software 17.0.

## Results and discussion

### Chemical variability of essential oils according to population altitude

Seven wild populations of *A. herba-alba* were collected during the flowering phase and from different altitudes which ranged from 194 m (population 1) to 841 m (population 7) (Table 1). The analyzed populations were located in the same region (Baten Zamour, south west of Tunisia) with an inferior arid climate characterized by an average annual rainfall of 150 mm/year. The chemical composition of essential oils was investigated using both GC/FID and GC/MS techniques. The percentages and the retention indices of the identified compounds of these essential oils were listed in Table 2 in the order of their elution on the HP-5MS column. Seventy-five compounds, representing 92.0 to 96.7% of the total essential oils, were identified and separated on the basis of their chemical structures into 5 classes (Table 2). Haouari and Ferchichi ([2009]) identified one hundred compounds in essential oils of subcultured *A. herba-alba* originated from different localities in sub-arid to Saharan domains of Tunisia. By contrast, the present study showed that twenty two compounds were not previously reported by Haouari and Ferchichi ([2009]) which strengthen the idea of the large chemical polymorphism of *A. herba-alba* essential oils. Whatever the altitude level, all these essential oils were characterized by very high percentage of monoterpenes (58.7–81.3%) and especially the oxygenated ones (52.1–72.6%) which constituted the predominant class as was found previously for *A. herba-alba* essential oils (Boukrich et al. [2010]; Zouari et al. [2010]). The sesquiterpenes were also represented mainly by oxygenated sesquiterpenes (4.4–14.5%). Nevertheless, Haouari and Ferchichi ([2009]) identified some populations of *A. herba-alba* from Southern Tunisia, where sesquiterpenes were more abundant than monoterpenes.

**Table 2:** **Mean percentage of compounds (%) in** ***A. herba-alba*** **essential oils**

No^a^	Compounds	RI^b^	1^c^	2^c^	3^c^	4^c^	5^c^	6^c^	7^c^	***p***_1_Fisher test	***p***_2_Kruskal- Wallis test
1	*cis*-Salvene	848	0.1					0.1	0.1	na	ns
2	Tricyclene	919	0.3		0.1	0.1	0.1	0.1	0.1	ns	ns
3	α-Thujene	924	0.2	0.4	0.6			0.1	0.1	na	ns
4	α-Pinene	930	1.6	1.9	3.2	1.9	2.7	1.4	1.7	ns	ns
5	Camphene^e^	946	2.8	0.4	0.8	0.8	0.8	0.5	0.9	*	ns
6	Verbenene	950	0.3	0.3	0.3	0.2	0.3	0.2	0.4	ns	ns
7	2(5H)-Furanone, 5,5-dimethyl-	952							0.1	na	ns
8	Sabinene	970	1.2	0.6	0.3	0.3	0.3	0.9	0.7	ns	ns
9	3-Octenol	972			0.2					na	ns
10	β-Pinene^e^	973	0.3		0.1		0.1		0.1	na	*
11	Myrcene^e^	986	0.2	0.4	0.5	0.5	0.4	0.6	0.9	*	*
12	Benzene,1,2,4-trimethyl-	990	0.3	0.3	0.4	0.8	0.3	0.4	0.6	ns	ns
13	α-Phellandrene	1000			0.1	0.1	0.1	0.1	0.1	na	ns
14	o-Isopropenyltoluene	1009	0.1	0.1	0.3	0.2	0.1	0.1	0.1	ns	ns
15	α-Terpinene	1013	0.1	0.2	0.1	0.1	0.2	0.3	0.3	ns	ns
16	p-Cymene^e^	1021	2.6	1.3	1.3	1.8	1.5	1.6	2.1	ns	*
17	1,8-Cineole	1028	4.1	2.5	1.6	2.3	3.5	1.5	1.9	ns	ns
18	γ-Vinyl-γ-valerolactone	1038	0.2	0.1	0.1	0.2	0.2	0.2	0.4	ns	ns
19	*cis*-Arbusculone	1049	0.7	3.2	3.0	2.2	3.0	1.6	2.8	ns	ns
20	γ-Terpinene	1055	0.2	0.4	0.2	0.3	0.3	0.4	0.5	ns	ns
21	*trans*-Arbusculone	1067	0.6	2.4	2.1	1.6	2.1	1.3	2.2	ns	ns
22	Terpinolene	1085	0.1	0.2	0.3	0.3	0.3	0.2	0.3	ns	ns
23	Linalool	1095		0.1	0.1	0.2	0.1		0.1	na	ns
24	Filifolone	1101	1.2	1.0	2.2	2.4	1.9	1.0	1.6	ns	ns
**25**	**α-Thujone** ^**d**^	1102	**13.0**	**11.4**	0.5	2.1	2.6	**20.2**	**8.2**	ns	ns
**26**	**β-Thujone** ^**d**^	1117	**9.2**	4.7	1.3	1.9	2.7	**9.9**	3.5	ns	ns
27	*cis*-2-p-Menthen-1-ol	1119		0.1						na	ns
**28**	**Chrysanthenone** ^**d**^	1124	4.7	**7.7**	**14.0**	**14.0**	**9.9**	**8.3**	**10.9**	ns	ns
29	*trans*-2-p-Menthen-1-ol	1135		0.1						na	ns
30	1-Terpineol	1138	0.7	1.5		0.8		0.1	0.1	na	ns
31	Pinocarveol^e^	1139		1.0	5.8	2.7	4.3	3.0	3.1	***	**
**32**	**Camphor** ^**d,e**^	1147	**10.7**	0.7	1.2	0.4	0.7	0.6	3.4	**	**
33	Sabina ketone	1158	0.3	0.1	1.9	1.0	1.2	0.8	0.4	ns	ns
34	Pinocarvone	1163	1.3	1.2	1.1	0.9	1.3	0.3	1.2	ns	ns
35	Borneol	1167	1.4	0.5	0.8	0.6	0.7	0.4	0.8	ns	ns
36	4-Terpineol	1177	1.5	0.9	0.7	0.6	0.8	1.0	1.0	ns	ns
37	p-Cymen-8-ol	1183	0.4	0.2	0.4	0.3	0.3	0.3	0.4	ns	ns
38	α-Terpineol^e^	1189	0.3	0.1						na	*
39	Myrtenol	1194	0.4	0.2	0.5	0.1	0.4	0.5	0.5	ns	ns
40	Verbenone	1208	0.3	0.4	0.6	0.8	0.4	0.4	0.8	ns	ns
41	Carveol	1217		0.2	0.2	0.3	0.2	0.3	0.2	ns	ns
42	Nordavanone	1225	0.7	0.5	0.1	1.4	0.4	0.2	0.4	ns	ns
43	Cuminal	1237	0.4	0.2	0.1	0.1	0.2	0.3	0.3	ns	ns
44	Piperitone	1252	0.3	0.3	0.4	0.3	0.2	0.6	0.5	ns	ns
**45**	**Chrysanthenyl acetate** ^**d,e**^	1257	**10.2**	**7.9**	**21.1**	**11.5**	**18.7**	**9.1**	**8.2**	*	*
46	Isopiperitenone	1270	0.4	0.2	0.3	0.5	0.3	0.2	0.4	ns	ns
47	Bornyl acetate	1281	1.4	0.2	0.4	0.2	0.5	0.2	0.5	ns	ns
**48**	**Sabinyl acetate** ^**d**^	1290	**7.7**	**10.8**	2.8	4.0	6.2	**10.3**	**10.6**	ns	ns
49	Thymol	1301	0.5	0.3	0.6	0.4	0.3	0.6	0.6	ns	ns
50	Myrtenyl acetate	1318	0.7	0.6	0.7	1.2	0.9	1.1	1.9	ns	ns
51	Piperitenone	1344	0.1	0.6	0.1	0.7	0.2	0.6	1.0	ns	ns
52	Norchrysanthemic acid methyl ester	1359	0.5	1.1	2.6	2.8	2.0	1.9	2.0	ns	ns
53	α-Copaene	1372	2.4	0.4	0.4	0.2	0.2	0.4	0.7	ns	ns
54	Methyl cinnamate	1379							0.3	na	ns
55	*cis*-Jasmone	1395	3.5	3.4	2.9	3.3	2.2	2.2	2.8	ns	ns
56	Davana furan	1399		0.1		0.2	0.1	0.1	0.1	na	ns
57	β-Caryophyllene	1417	0.2	0.4	0.4	0.3	0.5	0.1	0.4	ns	ns
58	p-Nitroanisole	1450	0.2	1.2	1.5	1.9	1.1	0.4	0.9	ns	ns
59	Aromadendrene	1458	0.2	1.1	0.5	1.5	0.8	0.5	0.6	ns	ns
60	α-Amorphene	1472	0.2	0.2	0.2	0.6	0.6	0.3	0.4	ns	ns
61	Germacrene-D^e^	1478	0.8	2.2	1.4	2.4	2.2	1.8	1.4	**	*
62	Bicyclogermacrene^e^	1493	0.4	1.7	0.7	1.3	1.1	0.9	0.9	***	*
63	Davana ether (isomer)^e^	1496	0.1	1.0	0.6	1.5	1.0	0.4	0.4	**	*
64	Davana ether (isomer)^e^	1504	0.2	2.5	0.7	3.4	2.1	0.9	0.7	*	*
65	Davana ether (isomer)	1514		0.9	1.2	0.9	0.6	0.3	0.2	na	ns
66	δ-Cadinene	1518	0.2	0.4		0.4	0.6	0.2	0.1	ns	ns
67	Nerolidol	1556	0.4	0.2		0.1	0.4	0.1	0.3	na	ns
68	Palustrol	1567		0.2	0.1					na	ns
69	Spathulenol^e^	1579	2.4	3.0	3.4	3.0	3.0	1.6	1.8	*	*
70	Caryophyllene oxide	1588	0.4	0.9	1.1	0.8	0.9	0.4	0.7	ns	ns
71	Viridiflorol	1593	0.2	1.0	0.3	0.4	0.2		0.1	na	ns
72	*cis*-Davanone^e^	1608	0.3	1.3	2.1	1.2	2.6	0.7	2.8	**	**
73	Isospathulenol^e^	1637	0.2	1.7	1.2	1.4	1.3	0.5	1.1	**	*
74	t-Cadinol	1653	0.1	0.3		0.3		0.1		na	ns
75	Vulgarol B^e^	1688		0.8		1.0	0.6	0.3	0.3	***	**
	Total identified (%)		96.7	94.4	94.8	92.0	95.8	96.0	96.0		
	Grouped components (%)										
	Monoterpene hydrocarbons		10.0	6.2	8.3	6.6	7.1	6.5	8.2		
	Oxygenated monoterpenes		71.3	56.6	60.2	52.1	59.2	72.6	64.0		
	Sesquiterpene hydrocarbons		4.3	6.1	3.5	6.3	5.4	4.0	4.5		
	Oxygenated sesquiterpenes		4.4	14.2	10.6	14.5	13.3	5.5	8.3		
	Others		6.7	11.3	12.2	12.5	10. 8	7.4	11.0		

^a^The numbering refers to elution order of compounds from a HP-5MS column and their percentages were obtained by FID peak-area normalization. The percentage for each population represents the average calculated on five individuals (*n* = 5). ^b^RI, retention indices calculated against C_7_-C_25_ n-alkanes mixture on the HP-5MS column. ^c^For the detailed description of the populations (1–7) locations, see Table 1. ^d^Major compound in bold fond. ^e^Compounds with a statistically significant variation among altitudinal populations. *p*_1_: *p*-values using Fisher test (one-way analysis of variance) applied for normally distributed variables. Fisher test was not applicable (na) for non-Normal distributed variables. *p*_2_: *p*-values using non-parametric Kruskal-Wallis test (one-way analysis of variance). *p*_1_ or *p*_2_ are extremely significant (***) at *p* ≤ 0.001, highly significant (**) at 0.001 ≤ *p* ≤ 0.01, significant (*) at 0.01 ≤ *p* ≤ 0.05 and not significant (ns) at *p* > 0.05.

Results showed that Fisher test was not applicable for 18 essential oil compounds (non-Normal distributed variables) (*P*_1_, Table 2). Therefore, for these compounds having skewed distributions, a non parametric one-way analysis of variance Kruskal-Wallis test was performed (*P*_2_, Table 2). By combining the two tests, the analysis of variance showed that altitude presented a significant effect on the variation of the concentration of 16 over 75 compounds. Table 2 also showed that the major compounds (>7%) were α-thujone **25**, β-thujone **26**, chrysanthenone **28**, camphor **32**, chrysanthenyl acetate **45**, and sabinyl acetate **48**. However, they could not be considered as major compounds in all altitudinal populations. Results showed that chrysanthenone **28** (7.7–14.0%) and chrysanthenyl acetate **45** (7.9–21.1%) were highly represented in most populations. α-Thujone **25** (8.2–20.2%) and sabinyl acetate **48** (7.7–10.8%) characterized four populations. β-Thujone **26** characterized population 1 (9.2%) and population 6 (9.9%). Whereas, camphor **32** (10.7%) characterized only population 1. Furthermore, when taking into account α-thujone **25** and β-thujone **26**, essential oils of populations 1 and 6 were dominated by thujones which ranged from 22.20 to 30.1%. Previous works on *A. herba-alba* showed an important intraspecific chemical polymorphism of the essential oils among samples belonging to different geographical regions and climatic conditions. In fact, with a tremendous variability, α-thujone (not detected–80%), β-thujone (not detected–58%), chrysanthenone (not detected–65%), camphor (not detected–70%), and 1,8-cineole (not detected–28%) were reported in most studies from all the countries, where *A. herba-alba* grows. In addition, with a more restricted occurrence, davanone (not detected−51%) and *cis*-chrysanthenyl acetate (not detected–69%) showed high variability in *A. herba-alba* from Spain and Tunisia (Belhattab et al. [2014]). Usually one of these seven compounds or some of them in different proportions, dominated *A. herba-alba* essential oils, which is in agreement with the results obtained for populations 3, 4 and 5 (Table 2). However, essential oils of populations 1, 2, 6, and 7 contained sabinyl acetate **48** at relatively high percentages (7.7–10.8%) seems to be characteristic to Southern Tunisian *A. herba-alba* as previously described (Haouari and Ferchichi [2009]; Boukrich et al. [2010]; Zouari et al. [2010]).

Although all the essential oils analyzed in the present study could be classified as oxygenated monoterpene-rich oils, they showed wide range of variation in all their compounds. Therefore, the chemical differentiation among populations assessed by linear discriminant and cluster analyses was carried out.

### Chemical clusters among altitudinal populations

**Figure 1:**
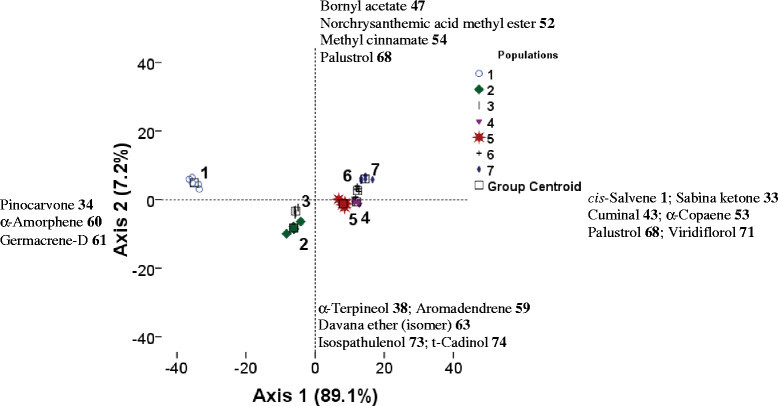
**Linear discriminant analysis (LDA) for the essential oil compounds of altitudinal** ***A. herba-alba*** **populations.** The average contents of the essential oil compounds were projected onto the first two principal axes (+ and - indicate positive and negative correlations with the axes, respectively). Coding numbers of altitudinal populations: see Table 1.

To identify possible relationships between volatile compounds and altitudinal populations, linear discriminant analysis (LDA) was applied (Figure 1). The LDA, performed on average contents of all compounds for each population showed that the first two principal axes represented 96.30% of the total variation. Moreover, principal axes of Figure 1 showed that essentially minor compounds played an important role to discriminate between individuals. The plot of the projection of the values of all the compounds onto the first two principal axes revealed 3 population groups associated with altitudinal levels (Figure 1). The first group, represented by population 1 (194 m altitude), was situated at the periphery of the plot. The second group, represented by populations 2 and 3 (206–219 m altitude), was situated in the centre of axes 1 and 2 at their negative sides. While, the third group represented by populations 4, 5, 6, and 7 (230–841 m altitude), was situated at the positive side of axis 1.

**Figure 2:**
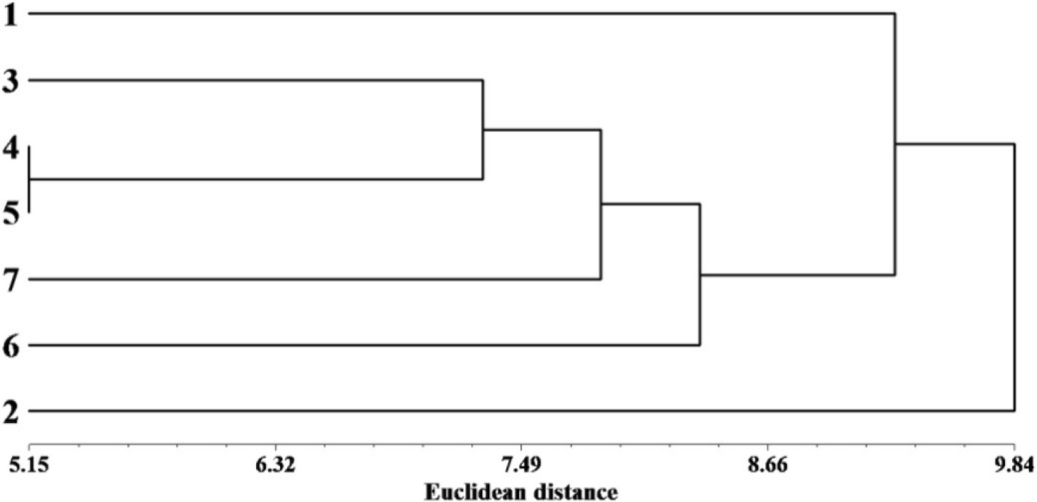
**Dendrogram obtained by cluster analysis based on Euclidean distance performed on the essential oil compounds of altitudinal** ***A. herba-alba*** **populations.** Coding numbers of altitudinal populations: see Table 1.

In addition to the linear discriminant analysis and to better characterize populations groups, cluster analysis (dendrogram) was applied to a matrix linking essential oil composition to altitude population (Figure 2). The dendrogram generated from the Euclidean distances performed on the essential oils compounds of *A. herba-alba* populations showed population groupings globally similar to those observed by the LDA clustering. Figure 2 showed that the studied populations, which were geographically close populations, were separated in three main clusters. The first and the second groups were represented by populations 1 and 2, respectively. While, the third cluster was represented by populations 3, 4, 5, 6, and 7 (Figure 2).

In the case of the genus *Artemisia*, the altitudinal variation of the volatile compounds of some *Artemisia* species was previously studied. Haider et al. ([2010]) reported the essential oil composition of *Artemisia nilagirica* growing at different altitudes in Himachal Pradesh from India. The major compounds of the analyzed essential oils showed important variation with changes in altitude. At lower, middle, and higher altitudes, the major compounds were caryophyllene oxide (28.6%), borneol (35.8%), and camphor (46.9%), respectively (Haider et al. [2010]).

### Influence of population altitude on yield, metal (Fe^2+^) chelating and DPPH radical-scavenging activities of essential oils

The essential oils were extracted by hydrodistillation from the dried aerial parts of *A. herba-alba* collected during the flowering stage and their yields ranged from 1.30 to 2.80% (v/w) (Table 3). These yields were higher in populations of high altitudes (populations 6 and 7) with a maximum obtained in the population 6 located at 361 m. Nevertheless, it was reported that the lowest yield of *Artemisia roxburghiana* essential oil was obtained from plants collected at higher altitudes (Haider et al. [2009]). It was also reported that *Thymus praecox* growing in higher altitudes contained lower essential oil than those grown in lower altitudes (Avci [2011]). The altitude, the edapho-climatic conditions, as well as the phase of the plant development induce high variations on yield and chemical composition of essential oils according to the plant species (Rahimmalek et al. [2009]; Zouari et al. [2012]).

**Table 3:** **Yield, and scavenging and chelating activities of** ***A. herba-alba*** **essential oils**

Populations	Oil yield	Scavenging activity	Chelating activity
1	2.42 ± 0.01^bc^	10.60 ± 2.56^a^	0.28 ± 0.12^ab^
2	1.73 ± 0.43^ab^	13.80 ± 4.99^a^	0.47 ± 0.13^bc^
3	1.44 ± 0.10^a^	10.42 ± 2.22^a^	0.14 ± 0.01^a^
4	1.36 ± 0.17^a^	15.30 ± 3.60^a^	0.75 ± 0.13^d^
5	1.30 ± 0.18^a^	15.46 ± 4.02^a^	0.67 ± 0.08^cd^
6	2.79 ± 0.69^c^	11.74 ± 2.58^a^	0.66 ± 0.21^cd^
7	2.67 ± 0.80^c^	9.76 ± 2.68^a^	0.56 ± 0.09^cd^

Values represent mean ± standard deviation (*n* = 5). Values followed by the same letter under the same row, are not significantly different (*p* > 0.05). Oil yield was expressed in ml/100 g dry matter. DPPH radical-scavenging activity (%) was determined for essential oil concentration at 1 mg/ml. Metal (Fe^2+^) chelating activity was presented as IC_50_ values (mg/ml). Coding numbers of altitudinal populations: see Table 1.

After that, essential oils were subjected to screening for their metal (Fe^2+^) chelating and DPPH radical-scavenging activities (Table 3). Results of the present study confirmed that *A. herba-alba* essential oils were not able to reduce effectively the stable free radical DPPH as compared to the synthetic antioxidant BHA. In fact, DPPH radical-scavenging activity of essential oils at 1 mg/ml were lower than 15.46% as was found previously for other *Artemisia* species essential oils (Lopes-Lutz et al. [2008]), whereas the IC_50_ value of the synthetic antioxidant BHA was 13 μg/ml. Metal chelating activity was known as one of antioxidant mechanisms, since it reduced the concentration of the catalyzing transition metal in lipid peroxidation. Among the transition metals, Fe^2+^ ion was known as the most important lipid oxidation prooxidant due to its high reactivity (Liu et al. [2007]). Results showed that statistically significant differences of chelating activity were affected by the population altitude. In fact, the highest chelating activity (IC_50_ value = 0.14 mg/ml) was found in population 3 located at 219 m. Although the chemical EDTA (IC_50_ value = 40 μg/ml) presented the highest chelating activity, natural compounds were of growing interest as compared with synthetic ones.

### Influence of population altitude on antimicrobial activities of essential oils

**Figure 3:**
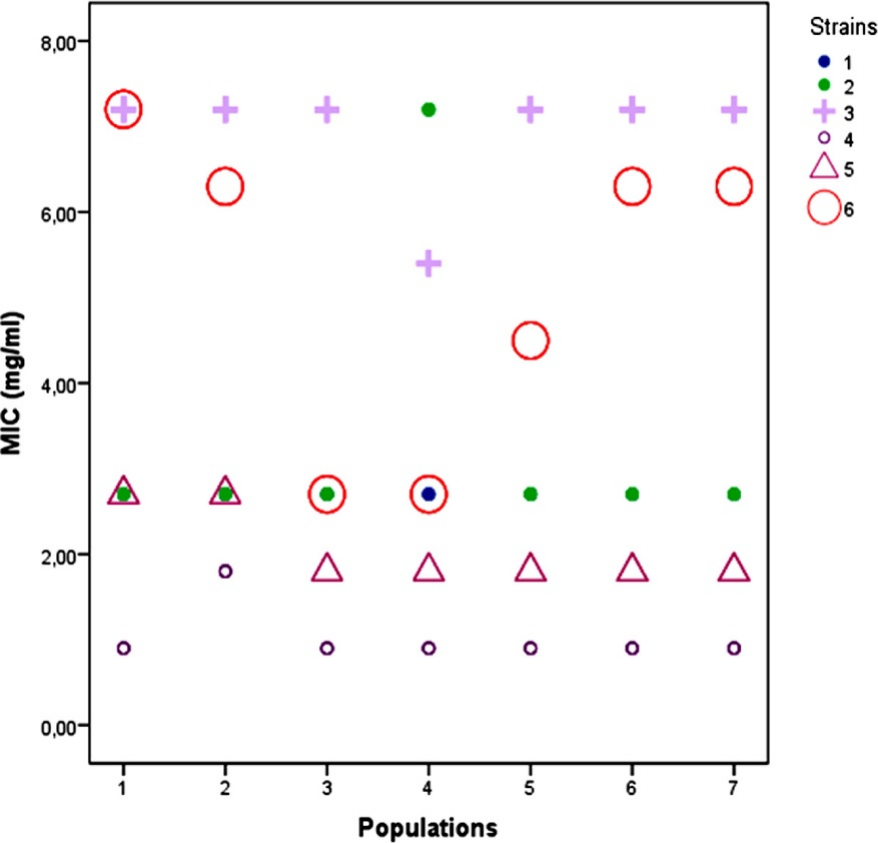
**Distribution of minimum inhibitory concentrations (MIC, mg/ml) of essential oils from altitudinal** ***A. herba-alba*** **populations against six bacterial strains.** The analyzed strains were: 1: *Klebsiella pneumoniae*, 2: *Escherichia coli*, 3: *Salmonella typhimurium*, 4: *Enterococcus faecalis*, 5: *Bacillus pumulis*, and 6: *Micrococcus luteus*. Coding numbers of altitudinal populations: see Table 1.

The antimicrobial activities of *A. herba-alba* essential oils against six species of microorganisms was assessed by the determination of minimum inhibitory concentrations (MICs) values (mg of oil/ml of medium). As it can be seen from Figure 3, *A. herba-alba* essential oils showed varying degrees of antimicrobial activities against all strains tested. In fact, the highest bacteriostatic activity was observed against *E. faecalis* (MIC = 0.9-1.8 mg/ml). However, *S. typhimurium* showed the less sensitivity (MIC of about 7 mg/ml). The determination of the correlation between the populations’ altitudes and all the measured MICs values (general model) showed no significant correlation (r = − 0.4, *p* = 0.57). Nevertheless, regression analysis showed that altitude variation was significantly correlated (r = 0.77) with MICs values of *M. luteus* following a cubic model (data not shown). The antimicrobial activity of *A. herba-alba* essential oils might be related to their oxygenated monoterpenes compounds which constituted about (52.1–72.6%) of the total oil as was previously suggested (Cox et al. [2000]; Lopes-Lutz et al. [2008]). In fact, it was shown that monoterpenes in essential oils are able to affect cellular integrity resulting in alteration in permeability of microbial cell and mitochondria membranes. Essential oils are also able to inhibit the synthesis of DNA, RNA, proteins, and polysaccharides in bacterial cells (José Abad et al. [2012]). Previous works focusing on the antimicrobial activities of different *Artemisia* essential oils tried to correlate these activities to one or many major compounds. However, it is difficult to attribute the antibacterial activity of a complex mixture to a particular compound. In fact, minor compounds as well as compounds with synergistic or antagonistic effects may play an important role in modulating the antibacterial activity of the entire essential oil. In this way, an essential oil is defined not only by its major compounds, but rather by a majority of all its compounds (Zouari [2013]).

## Conclusions

*A. saharae* oils were extracted by hydrodistillation of leaves and flowers of the plant collected from seven different altitudes of the Baten Zamour region (southwest of Tunisia). The highest essential oil yields (2.70-2.80%) were obtained for populations of high altitudes. Sabinyl acetate which was detected in some populations at relatively high percentages (7.7–10.8%) seems to be characteristic to Southern Tunisian *A. saharae*. Three population groups associated with altitudinal levels were distinguished. It is worthy to note that the most discriminating compounds of chemical groups were the minor ones. Based on the determination of the minimum inhibitory concentration, a low to a moderate antimicrobial activity according to oils was revealed against six bacteria tested. No significant correlation of antimicrobial activity and altitude variation was observed. Despite *A. saharae* oils were not able to reduce effectively the stable free radical DPPH, they showed relatively important metal (Fe^2+^) chelating activity. Altitude seems to be an important factor influencing the yield and the chemical profile of *Artemisia saharae* essential oils, which can be used as a criterion for selection harvesting area by relevant industries.

## Authors’ original submitted files for images

Below are the links to the authors’ original submitted files for images. Authors’ original file for figure 1 Authors’ original file for figure 2 Authors’ original file for figure 3
