# Supplementary figures and images for: Essential oil variation in wild populations of Artemisia saharae (Asteraceae) from Tunisia: chemical composition, antibacterial and antioxidant properties
Source: Bot Stud. 2014 Dec 10;55:76. doi: 10.1186/s40529-014-0076-0 (PMC5430380; doi:10.1186/s40529-014-0076-0)

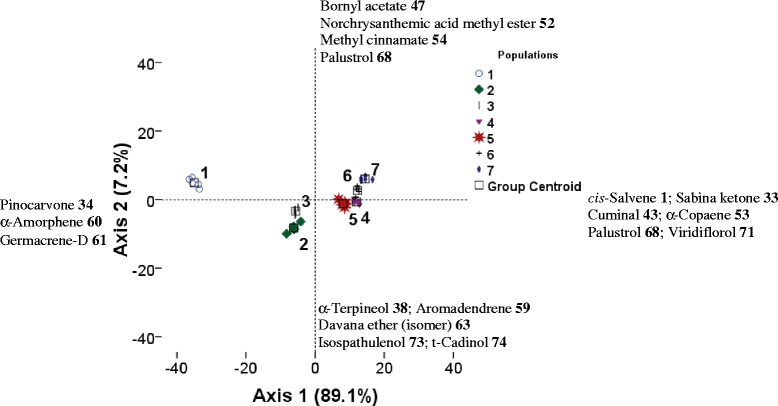

Supplement: Supplementary file 1 — Authors’ original file for figure 1 [file 40529_2014_9076_MOESM1_ESM.gif]

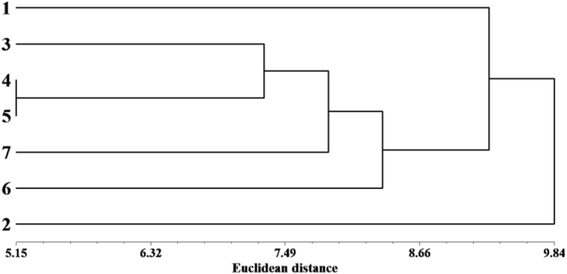

Supplement: Supplementary file 2 — Authors’ original file for figure 2 [file 40529_2014_9076_MOESM2_ESM.gif]

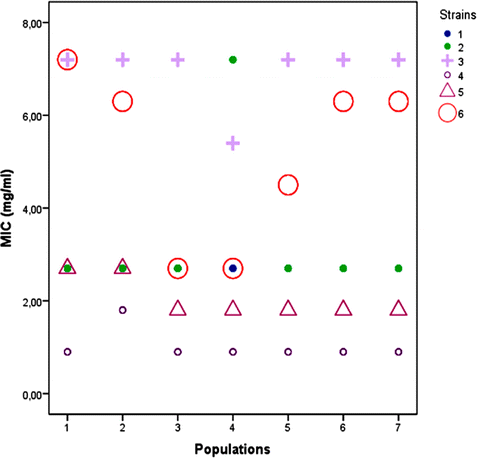

Supplement: Supplementary file 3 — Authors’ original file for figure 3 [file 40529_2014_9076_MOESM3_ESM.gif]
